# Pituitary hormone deficiencies in prolactinomas: prevalence, predictors, and functional recovery

**DOI:** 10.3389/fendo.2025.1705300

**Published:** 2025-11-10

**Authors:** Antonio Prinzi, Ginevra Fava, Ignazio Bacchi, Federica Spitali, Antonio Galvano, Giorgio Arnaldi, Francesco Frasca, Valentina Guarnotta, Pasqualino Malandrino

**Affiliations:** 1 Endocrinology Unit, Department of Clinical and Experimental Medicine, University of Catania, Garibaldi-Nesima Medical Center, Catania, Italy; 2 Department of Precision Medicine in Medical, Surgical and Critical Care (Me.Pre.C.C.), University of Palermo, Palermo, Italy; 3 Unit of Endocrinology, Department of Health Promotion, Mother and Child Care, Internal Medicine and Medical Specialties, Policlinico Paolo Giaccone, Università Degli Studi di Palermo, Palermo, Italy

**Keywords:** prolactinomas, pituitary adenoma, hypopituitarism, pituitary function recovery, neuroendocrine tumors

## Abstract

**Introduction:**

To evaluate the prevalence of pituitary hormone deficiencies in patients with prolactinomas, identify clinical and radiological predictors of non-gonadal hypopituitarism at diagnosis, and evaluate the potential for pituitary function recovery over long-term follow-up.

**Methods:**

We conducted a retrospective multicenter study including 145 patients with prolactinomas diagnosed between 2000 and 2024 at two tertiary centers. All anterior pituitary axes were evaluated at diagnosis and during follow-up.

**Results:**

At diagnosis, 54 of 145 patients (37.2%) had at least one pituitary hormone deficiency. Hypogonadism was the most common deficit (34.5%), followed by non-gonadal hypopituitarism in 14.5%, including secondary adrenal insufficiency: 8.3%, central hypothyroidism: 7.6%, growth hormone deficiency (GHD): 6.9%. Macroadenomas were significantly more prevalent than microadenomas (25.8% vs. 2.7%, p<0.001). Tumor size was the only independent predictor of non-gonadal hypopituitarism at diagnosis (OR: 1.1, 95%CI: 1.03–1.20; p=0.007). ROC analysis identified 17 mm as the optimal cut-off to predict non-gonadal pituitary hormone deficiencies at diagnosis (sensitivity 84%, specificity 77%, AUC = 0.836). During follow-up (median 70 months), 66.7% of patients recovered at least one pituitary axis, with higher recovery in microadenomas (100% vs. 63.0%, p=0.038). Tumor size remained the strongest predictor of recovery (OR: 0.56, 95%CI: 0.34–0.94; p=0.029).

**Conclusions:**

Non-gonadal hypopituitarism is common in prolactinomas, especially larger tumors. Tumor size was the strongest predictor of both the presence and recovery of hormonal deficits, with an optimal cut-off of 17 mm. Long-term follow-up is essential, as many patients, especially those with smaller tumors, recover pituitary function after treatment, with gonadal and adrenal axes showing the highest likelihood.

## 1 Introduction

Prolactin-secreting pituitary adenomas (prolactinomas), classified as pituitary neuroendocrine tumors (PitNETs), represent approximately 53% of all pituitary adenomas and originate from lactotroph cells (1). Similar to other pituitary adenomas, prolactinomas are classified based on tumor size into micro- (<10 mm) and macroprolactinomas (≥10 mm) (2). Microprolactinomas are more commonly diagnosed in women, whereas macroprolactinomas tend to occur more frequently in men (3). The clinical manifestations of prolactinomas may result from multiple mechanisms: the direct effects of elevated prolactin levels, the mass effect of the pituitary adenoma itself, which can cause headaches, visual field defects, or cranial nerve palsies and potential impairment of other anterior pituitary hormone axes (4, 5). The most frequent clinical features of prolactinomas result from hypogonadism, caused by hyperprolactinemia-induced suppression of gonadotropin-releasing hormone (GnRH) and pituitary gonadotropin secretion, likely mediated through inhibition of kisspeptin neurons (6). In women of reproductive age, menstrual disturbances occur in approximately 93% of cases, and galactorrhea is present in about 85% of these patients. In men, the typical symptoms include reduced libido and erectile dysfunction, while gynecomastia and galactorrhea are less frequent (1, 7). Medical treatment with dopamine agonists (DAs) is generally considered the first-line approach for both micro- and macroprolactinomas. Pituitary surgery, typically performed via the transsphenoidal route, is recommended in cases of DA-resistant prolactinomas, but may also be considered as a first-line option for microprolactinomas or well-circumscribed macroprolactinomas (Knosp grade 0 or 1), particularly when performed by an experienced neurosurgeon (3). While the impact of hyperprolactinemia on gonadal function is well established, much less is known about the prevalence and clinical significance of other pituitary hormone deficiencies in patients with prolactinomas (3). Several studies have reported that pituitary hormone deficiencies, beyond hypogonadism, are more frequently observed in patients with macroprolactinomas, with growth hormone deficiency (GHD) being the most common, followed by secondary hypothyroidism, and secondary adrenal insufficiency (8–13). Evidence also suggests that a substantial proportion of patients with hypopituitarism at diagnosis may recover pituitary function over time, particularly in response to DA therapy (8, 9, 12). However, existing studies are often limited by small sample sizes or restricted to highly selected populations, such as only male patients or those with macroprolactinomas. Therefore, the prevalence, predictors and extent of endocrine recovery remain poorly defined. The aim of this study was to evaluate the prevalence of pituitary hormone deficiencies at diagnosis in patients with prolactinomas, identify clinical and radiological predictors of hypopituitarism, and assess the potential for recovery of pituitary function during long-term follow-up.

## 2 Materials and methods

### 2.1 Study design and participant recruitment

In this retrospective multicenter study, we analyzed a cohort of patients with prolactinomas diagnosed at the Endocrinology Units of the Universities of Catania and Palermo, two tertiary referral centers in Italy. A total of 145 patients diagnosed with prolactinomas between 2000 and 2024 were included in this study. Diagnosis of prolactinoma was performed based on both the presence of hyperprolactinemia and signs and/or symptoms attributable to it, together with evidence of a pituitary adenoma on magnetic resonance imaging (MRI). Hyperprolactinemia was defined as plasma prolactin levels >3 times the upper limit of normal values (ULN) for the assay used, in two separate blood samples (14–16). In all centers, prolactin measurement was performed according to routine clinical practice, which included venous sampling with minimal stress conditions to avoid transient prolactin elevations (17). During the entire study period, serum prolactin concentrations were measured using automated chemiluminescent immunoassays. At the University of Palermo, the Immulite 2000 system (Diagnostic Products, Los Angeles, CA) was used up to 2015, and an electrochemiluminescence immunoassay (ECLIA) (Roche Diagnostics) thereafter. At the University of Catania, prolactin levels were consistently measured using the commercial kits from Abbott Laboratories (Abbott Park, Illinois, U.S.) throughout the study period. Patients with other causes of prolactin elevation (i.e.: renal failure, chronic use of antidopaminergic drugs, macroprolactin) were considered not eligible for the study, as well as patients with other causes of hypothyroidism, adrenal insufficiency, GHD, secondary hypogonadism and patients with other pituitary disorders including those with co-secreting pituitary adenomas (e.g., prolactin and GH). Patients with a history or radiological evidence of pituitary apoplexy were excluded from the study to avoid potential bias in the evaluation of pituitary function at diagnosis and during follow-up. The study was conducted in accordance with the Declaration of Helsinki and received approval from the Institutional Review Board and Ethical Committee Catania 2 in Sicily, Italy. Patient consent was waived due to the retrospective nature of the study.

### 2.2 Diagnosis, treatment and follow-up

At diagnosis, each patient was evaluated for the presence of clinical signs and symptoms of hypopituitarism. In addition, a gonadotroph, thyrotroph, corticotroph and somatotroph hormonal axes evaluation was performed. Hypopituitarism was defined as deficient secretion of one or more pituitary hormones diagnosed following the criteria of routine clinical practice. Secondary hypogonadism was defined as low or inappropriately normal levels of follicle-stimulating hormone (FSH) and luteinizing hormone (LH), in the presence of low testosterone in men (<8.0 nmol/L), or low estradiol (<200 pmol/L) with menstrual disturbances in premenopausal women. In postmenopausal women, hypogonadism was defined as gonadotropin levels below the expected postmenopausal reference range (18, 19). Secondary hypothyroidism was defined as a free thyroxine (FT4) level below the reference range in combination with a low or inappropriately normal thyroid-stimulating hormone (TSH) level (20). Secondary adrenal insufficiency was diagnosed when serum 08:00 h cortisol levels were low (<140 nmol/L) or when cortisol peaked at less than 500 nmol/L in response to synthetic ACTH stimulation (250 µg, i.v.) (21). GHD was established when serum insulin-like growth factor type 1 (IGF-1) concentration was lower than the age-specific lower limit of normal (22). Despite the long study period, the diagnostic criteria and references reported are based on the most recent international guidelines to maintain alignment with current endocrine standards and improve comparability with contemporary literature. All patients were treated with DAs. Of these, 140 patients received cabergoline as first-line therapy, starting at 0.5 mg once or twice weekly, with the dose progressively adjusted based on serum prolactin levels until normalization was achieved. Five patients initially received bromocriptine; in three cases treatment was later switched to cabergoline due to intolerance or suboptimal response, while two continued bromocriptine throughout follow-up. Following initiation of therapy, MRI was repeated after 3–6 months in patients with macroprolactinomas and after 12 months in those with microprolactinomas (3). Pituitary surgery was reserved for patients with resistance or intolerance to dopamine agonists, or in cases of neurological emergencies such as pituitary apoplexy or rapidly progressive visual impairment. Recovery of pituitary function was assessed at 6-month or annual intervals after initiation of DA therapy and at the last clinical evaluation. In addition, hormonal recovery was specifically reassessed at the time of DA withdrawal, when treatment discontinuation was achieved. Biochemical reassessment of each hormonal axis was performed as previously described.

### 2.3 Statistical analysis

Descriptive statistics were used to summarize the data. Continuous variables were expressed as mean ± standard deviation (SD) for normally distributed data, or as median and interquartile range (IQR; 25th–75th percentile) for non-normally distributed data. Categorical variables were reported as absolute numbers and percentages. Group comparisons for continuous variables were conducted using the Student’s t-test for normally distributed data, and the Mann–Whitney U test for non-normally distributed data. The chi-square (χ²) test was used to assess differences between categorical variables. To identify factors associated with non-gonadal hypopituitarism at diagnosis and with recovery of pituitary function during follow-up, univariate and multivariable logistic regression analyses were conducted. Results were expressed as odds ratios (ORs) with 95% confidence intervals (CIs). Variables with a p-value <0.05 in univariate analysis were included in the multivariable model, which was refined using a backward stepwise selection approach. The predictive performance of tumor diameter for non-gonadal hypopituitarism was assessed using receiver operating characteristic (ROC) curve analysis. The area under the ROC curve (AUC) with its 95% confidence interval was calculated to quantify discriminatory ability. A p-value < 0.05 was considered indicative of statistical significance. All statistical analyses were performed using STATA version 18.5 (Stata Corp LP, College Station, TX, USA).

## 3 Results

### 3.1 Clinical characteristics of patients with prolactinomas

**Table 1** summarizes the features of the 145 patients with prolactinomas at diagnosis. The mean age diagnosis was 36.9 ± 14.1 years. The median time to prolactin level normalization after initiating therapy was 21.07 months (25^th^–75^th^ IQR: 9.5–25.5 months). Ninety-eight (67.6%) patients were female and 47 (32.4%) were males. Median tumor size at diagnosis was 9.0 mm (25th–75th IQR = 5.0–19.0). Most patients had a microadenoma (79 patients, 54.5%), while 66 patients (45.5%) presented with a macroadenoma. Microadenoma were more frequent in female (72 out of 98, 73.5%) than male patients (7 out of 47, 14.9%; p<0.001). Median prolactin serum level was 168.6 ng/mL (25^th^-75^th^ IQR = 98.0–470.0). In most cases (78 patients, 54.2%), the diagnosis of prolactinoma was prompted by signs and symptoms related to hyperprolactinemia, in 28 patients (19.4%) the diagnosis was incidental, with no clinical features attributable to hyperprolactinemia or mass effect, in 23 patients (16.0%) the diagnosis followed symptoms caused by the tumor’s mass effect, while in 15 patients (10.4%) both hyperprolactinemia-related and mass effect-related symptoms contributed to the diagnosis. Accordingly, the majority of patients had a localized adenoma (109 patients, 75.2%). In 12 patients (8.3%), the tumor compressed the optic chiasm, in 13 patients (9.0%), there was radiological evidence of invasion of one or more cavernous sinuses, and in a smaller proportion (11 patients, 7.6%), the tumor both compressed the optic chiasm and invaded the cavernous sinuses. Consequently, only 20 patients (13.8%) presented with visual field defects at the time of diagnosis. Regarding treatment, 130 patients (89.7%) were managed with cabergoline alone, 13 patients (9.0%) received a combination of cabergoline and surgery, and only 2 patients (1.4%) required additional adjuvant therapy with gamma knife radiosurgery. Patients were followed up for a median time of 70.0 months (25^th^-75^th^ IQR = 28.3–133.6).

**Table 1:** Clinical features at diagnosis in patients with prolactinomas.

Features	Prolactinomas (N = 145)
Sex, n. (m/f)	47/98
Age at diagnosis (years; mean ± SD)	36.9 ± 14.1
Adenoma size (mm; median [IQR])	9.0 (5.0-19.0)
Prolactin serum (ng/mL; median [IQR])	168.6 (98.0-470.0)
Non-invasive adenoma, n. (%)	109 (75.2)
Optic chiasm compression, n. (%)	12 (8.3)
Cavernous sinus invasion, n. (%)	13 (9.0)
Optic chiasm and cavernous sinus invasion, n. (%)	11 (7.6)
Median Follow-up, (months; median [IQR])	70.0 (28.3-133.6)
Clinical presentation	
Hyperprolactinemia, n. (%)	78 (54.2)
Asymptomatic, n. (%)	28 (19.4)
Mass effect, n. (%)	23 (16.0)
Mass effect and hyperprolactinemia, n. (%)	15 (10.4)
Visual defects, n. (%)	20 (13.8)
Treatment	
Cabergoline, n. (%)	130 (89.6)
Cabergoline plus surgery, n. (%)	13 (9.0)
Cabergoline plus surgery and SRS, n. (%)	2 (1.4)

### 3.2 Hypopituitarism at diagnosis

At diagnosis, 54 of 145 patients (37.2%) had at least one pituitary hormone deficiency (**Table 2**). Hypogonadism was the most common defect, occurring in 50 patients (34.5%). Non-gonadal hypopituitarism was found in 21 patients (14.5%), most frequently as secondary adrenal insufficiency (12 patients, 8.3%), followed by central hypothyroidism (11 patients, 7.6%) and GHD (10 patients, 6.9%). Hypogonadism was significantly more frequent in patients with macroprolactinomas compared to those with microprolactinomas (60.6% vs. 12.7%, p<0.001), and non-gonadal hypopituitarism was also significantly more prevalent in macroadenomas (25.8% vs. 2.7%, p<0.001) than microadenomas. Moreover, hypogonadism was more frequent in male than in female patients (70.2% vs 17.4%, p<0.001). Notably, macroadenomas compared to microadenomas, were associated with higher rates of secondary adrenal insufficiency (13.6% vs. 3.8%, p=0.03), secondary hypothyroidism (13.6% vs. 2.5%, p=0.01), and GHD (13.6% vs. 1.3%, p=0.004). Secondary adrenal insufficiency and hypothyroidism were more frequent in males than females (19.2% vs. 3.1% and 19.2% vs. 2.0%, respectively; p<0.001), while GHD rates did not differ significantly between males and females (12.8% vs. 4.1%; p=0.053). When stratifying patients by tumor size, a significant progressive increase in the prevalence of non-gonadal hypopituitarism was observed across categories: 2.4% in tumors <5 mm, 3.1% in tumors 6–9 mm, 6.7% in tumors 10–14 mm, 17.6% in tumors 15–19 mm, and 38.2% in tumors >20 mm (p<0.001) (data not shown). A similar trend was observed for secondary hypogonadism, with a prevalence of 4.8%, 15.6%, 46.7%, 52.9%, and 70.6% across the same tumor size categories, respectively (p<0.001). The same was true for secondary adrenal insufficiency (2.4%, 0%, 0%, 2.0%, and 7.0%; p=0.005), secondary hypothyroidism (0%, 3.1%, 6.7%, 5.9%, and 20.6%; p=0.01) and GHD (0%, 0%, 6.7%, 11.8%, and 20.6%; p<0.001). Several clinical and radiological features were significantly associated with the presence of non-gonadal hypopituitarism at diagnosis. These included male sex (OR: 5.5, 95%CI: 2.04–14.86; p=0.001), tumor size (OR: 1.1, 95%CI: 1.05–1.14; p<0.001), compression of the optic chiasm and/or invasion of the cavernous sinuses (OR: 1.7, 95%CI: 1.13–2.58; p=0.011), presence of visual field defects at diagnosis (OR: 5.74, 95%CI: 1.98–16.63; p=0.001), and prolactin levels at diagnosis (OR: 1.0, 95%CI: 1.00–1.00; p=0.001). In the multivariable analysis, only tumor size remained independently associated with non-gonadal hypopituitarism at diagnosis (OR: 1.1, 95%CI: 1.03–1.20; p=0.007) (**Table 3**). Finally, ROC curve analysis was performed to evaluate the ability of tumor diameter to predict non-gonadal pituitary hormone deficiencies at diagnosis. A threshold of 17 mm was identified as the optimal cut-off, providing a sensitivity of 84% and a specificity of 77%, with excellent diagnostic performance (AUC = 0.836).

**Table 2:** Pituitary function at diagnosis and function recovery during follow-up in micro- (n. = 79 patients) and macroadenomas (n. = 66 patients).

Pituitary deficit	At diagnosis		P value	Function recovery (*)		P value
	Microadenoma	Macroadenoma		Microadenoma	Macroadenoma	
	n. (%)	n. (%)		n. (%)	n. (%)	
Secondary hypogonadism	10 (12.7)	40 (60.6)	<0.001	7 (63.6)	24 (66.7)	0.85
Secondary adrenal insufficiency	3 (3.8)	9 (13.6)	0.03	2 (66.7)	3 (33.3)	0.31
Secondary hypothyroidism	2 (2.5)	9 (13.6)	0.01	0 (0)	3 (66.7)	0.09
Growth hormone deficiency	1 (1.3)	9 (13.6)	0.004	0 (0)	1 (11.1)	0.72

*Data refers to patients with follow-up length of 12 months or more.

**Table 3:** Cox regression analysis to evaluate factors associated with non-gonadal hypopituitarism in patients with prolactinomas.

Features	Univariate analysis			Multivariable analysis		
	Odds ratio	95% CI	P value	Hazard ratio	95% CI	P value
Male sex	5.51	2.05-14.9	0.001			
Age at diagnosis	1.00	0.98-1.04	0.657			
Adenoma size	1.10	1.05-1.14	<0.001	1.11	1.03-1.20	0.007
Adenoma invasion	1.71	1.13-2.58	0.011			
Visual fields defects	5.74	1.98-16.6	0.001			
Prolactin at diagnosis	1.00	1.00-1.00	0.001			

### 3.3 Recovery of pituitary function during follow-up

Among the 54 patients diagnosed with hypopituitarism at baseline, 36 patients (66.7%) experienced recovery of at least one pituitary axis during follow-up (**Table 2**). Hypogonadism resolved in 31 out of 50 patients (62.0%), secondary adrenal insufficiency in 5 out of 12 patients (51.7%), secondary hypothyroidism in 3 out of 11 patients (27.3%), and GHD in 1 out of 10 patients (10.0%). Moreover, during follow-up, 14 of 56 patients (25%) discontinued cabergoline after achieving both remission of hyperprolactinemia and tumor resolution. When stratifying patients by baseline tumor size, we found that patients with microadenoma showed higher rate of recovery of pituitary function compared to patients with macroadenoma (100% vs. 63.0%; p=0.038) (data not shown). Consequently, A significant progressive decrease in recovery rates was observed across tumor size categories: 100% in tumors <5 mm, 100% in tumors 6–9 mm, 87.5% in tumors 10–14 mm, 88.9% in tumors 15–19 mm, and 48.3% in tumors ≥20 mm (p=0.016). Although not statistically significant, patients treated only with dopamine agonists showed a higher recovery rate (73.3%) compared to those treated with combined medical and surgical therapy (45.5%) (p=0.076). Among the clinical, biochemical, and radiological variables analyzed, only baseline tumor size (OR: 0.92, 95%CI: 0.88-0.98; p=0.009) and the presence of optic chiasm compression and/or cavernous sinus invasion (OR: 0.56, 95%CI: 0.34-0.94; p=0.029) were significantly associated with recovery of pituitary function during follow-up (**Table 4**). At multivariable analysis, baseline tumor size remained the only independent predictor of endocrine recovery (OR: 0.56, 95%CI: 0.34-0.94; p=0.029).

**Table 4:** Cox regression analysis to evaluate factors associated with pituitary recovery during follow-up.

Features	Univariate analysis			Multivariable analysis		
	Odds ratio	95% CI	P value	Hazard ratio	95% CI	P value
Male sex	0.80	0.23-2.82	0.734			
Age at diagnosis	0.99	0.95-1.03	0.550			
Adenoma size	0.93	0.88-0.98	0.009	0.93	0.87-1.00	0.045
Adenoma invasion	0.57	0.34-0.94	0.029			
Visual fields defects	0.48	0.13-1.71	0.256			
Prolactin at diagnosis	1.00	1.00-1.00	0.117			

## 4 Discussion

Our study shows that non-gonadal hypopituitarism is not uncommon in patients with prolactinomas, affecting approximately 14.5% of cases at diagnosis. While hypogonadism remains the most frequent hormonal disorder, largely driven by hyperprolactinemia, our findings emphasize that larger tumors may impair other anterior pituitary axes through mass effect, leading to clinically relevant deficiencies such as secondary adrenal insufficiency, secondary hypothyroidism, and GHD. Differently from previous studies, which reported GHD as the most frequent non-gonadal pituitary deficit, followed by secondary hypothyroidism and secondary adrenal insufficiency, our findings revealed a different distribution pattern: in our cohort, secondary adrenal insufficiency was the most prevalent non-gonadal deficiency, followed by secondary hypothyroidism and GHD (8–10, 23). As expected and according with previous studies, non-gonadal pituitary hormone deficiencies were significantly more frequent in patients with macroadenomas compared to those with microadenomas (25.8% vs. 2.7%) (8). Moreover, we observed a clear and progressive increase in the prevalence with increasing tumor size, reaching 38.2% in tumors >20 mm, compared to only 2.4% in those <5 mm. In line with this finding, ROC curve analysis identified a threshold of 17 mm as the optimal cut-off to predict non-gonadal hypopituitarism, with strong diagnostic performance. This finding is clinically relevant, as it suggests that, in clinical practice, patients with prolactinomas of this size or larger should undergo a comprehensive assessment of all anterior pituitary axes at diagnosis, given their markedly increased risk of clinically significant hormonal deficiencies. Conversely, in tumors <17 mm, where the prevalence of non-gonadal hypopituitarism is low, a more selective hormonal evaluation may be considered in asymptomatic patients, although external validation of this cut-off in independent cohorts is warranted before its widespread adoption in clinical practice.

Several studies have attempted to characterize the prevalence of pituitary hormone deficiencies in patients with prolactinomas. However, most are limited by small sample sizes and selective populations, often including only male patients or exclusively those with macroprolactinomas. Moreover, in many of these studies, not all pituitary axes were systematically evaluated, further limiting the accuracy and comparability of the reported data.

In the study by Behan et al., only 3 out of 35 patients (8.6%) with microprolactinomas exhibited pituitary hormone deficiencies beyond hypogonadism (specifically, three had secondary adrenal insufficiency and one had GHD), confirming that clinically relevant hypopituitarism is rare in smaller tumors (11). Similar findings were reported by Colao et al., in which none of the patients with microprolactinomas presented hypopituitarism beyond hypogonadism. Likewise, in the study by Iglesias et al., only one patient with a microprolactinoma showed hypopituitarism, specifically GHD (8, 9). Conversely, Karavitaki et al. focused exclusively on patients with macroprolactinomas and reported a substantially higher prevalence of pituitary hormone deficits at diagnosis: hypogonadism in 90% of patients, GHD in 83%, TSH deficiency in 36%, and ACTH deficiency in 17% (12). Other studies, such as those by Tirosh et al. and Sibal et al., specifically examined male patients with macroprolactinomas (10, 13). Both highlighted hypogonadism as the most frequent deficiency (87.6% and 77%, respectively), followed by GHD (23.3% and not available), secondary hypothyroidism (18.2% and 41%), and secondary adrenal insufficiency (14.1% and 23%).

Another key finding of our study is the relatively high rate of recovery of pituitary function during follow-up, particularly among patients with smaller tumors. While hypogonadism was the most frequently reversible deficit (62%), recovery of other axes, though less frequent, was still observed, especially for secondary hypothyroidism (45.5%) and adrenal insufficiency (25%). Although a large body of literature has investigated the recovery of gonadal function following treatment of prolactinomas, reporting variable rates ranging from 26.7% to 79%, data on the reversibility of other pituitary axis remain scarce (23–26). Studies evaluating recovery of non-gonadal hypopituitarism have reported thyroid function recovery rates ranging from 12.5% to 44%, recovery of secondary adrenal insufficiency from 0% to 85.7%, and GHD recovery from 0% to 67% (10, 12, 13, 27). However, these studies were generally limited by very small sample sizes which significantly reduces the reliability and generalizability of their findings. By contrast, our study provides a more comprehensive evaluation of endocrine recovery in a larger and well-characterized cohort, allowing for a more accurate estimation of recovery rates and the identification of predictive factors Importantly, tumor size emerged as the strongest predictor of hormonal recovery, suggesting that the extent of initial damage or compression plays a crucial role in determining reversibility. Taken together, these findings indicate that tumor size exerts a dual influence on pituitary function acting as a determinant of both baseline hormonal deficits and the likelihood of subsequent recovery. Larger adenomas are more likely to cause irreversible damage to the normal gland, whereas smaller lesions often preserve sufficient residual function to allow restoration once decompression is achieved under DAs therapy. This dual impact reflects both the mechanical and temporal dimensions of tumor growth: chronic compression and ischemic changes in large prolactinomas may result in permanent loss of pituitary tissue, while smaller or more recently developed lesions are less likely to induce structural damage and may allow functional recovery once the mass effect is relieved.

From a clinical perspective, our findings highlight the importance of systematically assessing all anterior pituitary axes in patients with larger prolactinomas, given the significantly increased risk of non-gonadal hypopituitarism. Early identification of these deficiencies is crucial, as timely hormonal replacement can prevent serious morbidity. Moreover, the demonstration that a substantial proportion of patients, especially those with smaller tumors, recover pituitary function over time reinforces the value of careful longitudinal monitoring and tailored therapeutic strategies. Strengths and limitations of our study should be acknowledged. Strengths include the relatively large sample size, the comprehensive evaluation of all pituitary axes at diagnosis, and the long follow-up period, which allowed us to capture recovery patterns over time. Our study has several limitations. First, its retrospective design limits control over data collection and introduces potential biases related to missing or incomplete information. Second, the assessment of GHD was based solely on IGF-1 concentrations rather than dynamic testing, as the insulin tolerance test and glucagon stimulation test, considered gold standards for diagnosing GHD, were not systematically performed (22). Therefore, the true prevalence of GHD may have been underestimated or misclassified. Furthermore, although our ROC analysis identified a tumor size cut-off with strong predictive performance, external validation in independent cohorts is warranted. In addition, data regarding Knosp grade for tumor invasiveness and cystic morphology were not consistently available across centers and could not be systematically analyzed, representing an additional limitation. Finally, the lack of a control group of patients with non-functioning PitNETs limited our ability to determine whether the prevalence and recovery of non-gonadal hypopituitarism differ independently of hyperprolactinemia. Future studies directly comparing prolactinomas with non-functioning PitNETs are warranted to clarify this issue. Moreover, information on the median time to recovery of individual pituitary axes, the evolution of symptoms leading to diagnosis, and their relationship with prolactin normalization was not systematically collected.

## 5 Conclusions

Non-gonadal hypopituitarism is a relatively frequent finding in patients with prolactinomas, particularly in those with larger tumors. Tumor size is the strongest predictor both for the presence of hormonal deficits at diagnosis and for their potential recovery during follow-up, with a clinically relevant threshold identified at 17 mm. These findings highlight the need for a systematic and comprehensive assessment of all anterior pituitary axes in patients with macroprolactinomas to ensure timely recognition and management of hormone deficiencies. From a clinical perspective, integrating tumor size into the initial risk assessment may help identify patients who require closer endocrine monitoring and early hormone replacement, while also recognizing those with smaller, non-invasive tumors who are more likely to recover pituitary function after DA therapy. Longitudinal follow-up remains essential, as recovery of pituitary function can occur even after prolonged treatment, reinforcing the importance of individualized and dynamic patient management.

## Data Availability

The raw data supporting the conclusions of this article will be made available by the authors, without undue reservation.
